# DCE: A Distributed Energy-Efficient Clustering Protocol for Wireless Sensor Network Based on Double-Phase Cluster-Head Election

**DOI:** 10.3390/s17050998

**Published:** 2017-05-01

**Authors:** Ruisong Han, Wei Yang, Yipeng Wang, Kaiming You

**Affiliations:** School of Electronic and Information Engineering, Beijing Jiaotong University, Beijing 100044, China; hanruisong@bjtu.edu.cn (R.H.); yipeng@bjtu.edu.cn (Y.W.); youkaiming@bjtu.edu.cn (K.Y.)

**Keywords:** clustering, wireless sensor network, distributed, heterogeneous, energy consumption

## Abstract

Clustering is an effective technique used to reduce energy consumption and extend the lifetime of wireless sensor network (WSN). The characteristic of energy heterogeneity of WSNs should be considered when designing clustering protocols. We propose and evaluate a novel distributed energy-efficient clustering protocol called DCE for heterogeneous wireless sensor networks, based on a Double-phase Cluster-head Election scheme. In DCE, the procedure of cluster head election is divided into two phases. In the first phase, tentative cluster heads are elected with the probabilities which are decided by the relative levels of initial and residual energy. Then, in the second phase, the tentative cluster heads are replaced by their cluster members to form the final set of cluster heads if any member in their cluster has more residual energy. Employing two phases for cluster-head election ensures that the nodes with more energy have a higher chance to be cluster heads. Energy consumption is well-distributed in the proposed protocol, and the simulation results show that DCE achieves longer stability periods than other typical clustering protocols in heterogeneous scenarios.

## 1. Introduction

A wireless sensor network (WSN) consists of a large amount of tiny, inexpensive but smart sensors to perform distributed sensing tasks, and is the foundation of the Internet of Things (IoT). WSNs have received a great deal of interest in recent years due to their vast number of significant applications such as national security, military, and environmental monitoring [1]. It plays an important role in monitoring and analyzing the dynamic, hostile, unfamiliar and unexplored environment [2]. Since most of the wireless sensor nodes have a limited battery lifetime, constructing a large-scale and energy-efficient WSN is of great importance, but generally difficult to deal with.

**Figure 1:**
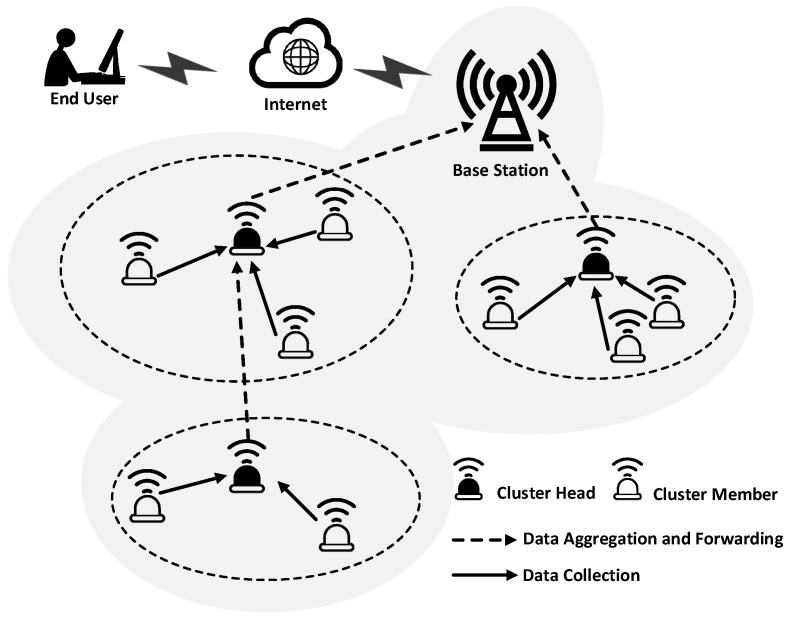
A clustered Wireless Sensor Network.

Topology control offers an effective approach to addressing these challenges in WSN. In general, topology control refers to a set of techniques that can reform the underlying network topology, aimed at system performance enhancements and/or cost reductions [3]. Among the techniques of topology control, clustering is an effective and widely used scheme for managing network topology. Illustrated by Figure 1, the sensor nodes in the WSN organize themselves into local clusters, in which the data sensed by cluster member (CM) nodes are aggregated by cluster head (CH), and then forwarded to the remote base station (BS). Since the correlation is strong between the data signals from nodes located close to each other, data aggregation can reduce the data set enormously, and present the end user with a high-level function of data [4]. Therefore, the network can achieve the effective data without wasting valuable energy and bandwidth resources transmitting all the data to the BS. Clustering in WSN has benefits like scalability, high energy efficiency, and reducing routing delay [5].

The last two decades have witnessed many clustering approaches being proposed for WSN, where energy conservation is the primary target. Generally, the operations of clustering protocols are divided into rounds. A round begins with a set-up stage which involves CH selection and cluster formation, and is then followed by a steady-state stage to perform data transmission. For a CH node, energy consumption is generally huge, since it has to collect, process and transmit all the data of its cluster. In order to maintain a well-distributed energy consumption and prolong the lifetime of WSN, the schemes for selecting proper CHs are of great importance, and regarded as the core of typical clustering algorithms. Although extensive research work has been conducted in the direction of CH selection, some basic problems in the design procedure still exist, such as neglecting the energy heterogeneity of sensor nodes, selecting inappropriate CH nodes, and poor scalability of centralized design. In the following text, we refer to the heterogeneous wireless sensor network as the WSN in which sensor nodes have different energy.

In this paper, we propose and evaluate DCE, a distributed energy-efficient clustering protocol based on a Double-phase Cluster-head Election scheme for heterogeneous wireless sensor network. In DCE, the procedure of CH election is divided into two phases. In the first phase, tentative cluster heads are elected based on probability, decided by the relative levels of initial and residual energy. Then, in the second phase, the tentative cluster heads are replaced by randomly-chosen high-energy cluster members to form the final CH set, if any member in their cluster has more residual energy. 

These two phases guarantee that the nodes with more energy a have higher chance of being the cluster heads, which contributes to maintaining well-distributed energy consumption in the proposed protocol. Moreover, the characteristics of heterogeneity and scalability are also taken into account. In brief, the main contributions of our work lie in that our double-phase cluster-head election scheme improves the possibility of avoiding choosing low-energy sensor nodes as cluster heads, and thus achieves better utilization of energy resources.

The remainder of this paper is organized as follows. We give a brief discussion of the related literature in Section 2. Section 3 introduces the heterogeneous network model, and presents some essential assumptions for our target problem. In Section 4, we present the DCE protocol in detail, and analyze its properties. Simulation work and numerical results are presented in Section 5, to show the effectiveness of DCE. Finally, we conclude the paper in Section 6.

## 2. Related Work

Clustering schemes can be classified using three main aspects, which are the properties of clusters, the capabilities of cluster heads, and the clustering process [6,7,8]. Extensive research work has been conducted by considering a single aspect or the combinations of various ones [9,10,11,12,13,14]. For the clustering process, methodology, objective, cluster head selection and algorithm complexity are the main classification methods. Our protocol is distributed in terms of methodology, and the objective is extending the stability period (the period of time until the first node depletes its energy) by adopting a double-phase cluster-head election scheme. Thus, the following review work centers on the clustering process of WSN.

Low Energy Adaptive Clustering Hierarchy (LEACH) [4,15] is one of most common clustering protocols in WSN. It is a self-organizing clustering protocol, which equalizes energy load distribution among sensor nodes by periodically rotating the cluster heads. In LEACH, each sensor node randomly elects itself to be a CH based on a pre-defined probability, and makes final decisions autonomously. The election probability is a periodic function of the operation round, which limits the epoch for a node to be the CH. Therefore, a periodical rotation of cluster heads can be used to balance the energy consumption. CH nodes broadcast announcements to all the nodes in the network, and the non-cluster-head nodes join in the nearest clusters, by comparing the strength of received signals from CH nodes. CH nodes take charge of making transmitting schedule, and receiving and aggregating the data from cluster members. Then, the aggregated data are forwarded back to the BS by single-hop communication. In brief, LEACH is concise and does not require large communication overheads and complicated control mechanisms. Unfortunately, since LEACH makes the assumption that all nodes start with equal energy, the performance of LEACH in heterogeneous WSN is not very good. Hence, the energy heterogeneity of sensor nodes should be considered when designing new protocols.

HEED, a hybrid, energy-efficient distributed clustering approach, was proposed in [16]. HEED periodically selects cluster heads, according to a hybrid of the residual energy of nodes and a second parameter like node proximity to its neighbors or node degree. Although HEED is capable of handling heterogeneous node batteries by adjusting the campaigning probability, it can’t avoid the situation where low-energy nodes have a larger probability of being the cluster heads than high-energy ones.

On the other hand, some heterogeneity-aware protocols such as SEP [17] and DEEC [18] are specially designed for heterogeneous WSN. SEP is aimed at prolonging the stability period of the two-level heterogeneous networks, which consist of two types of nodes according to the initial energy, i.e., normal nodes and the advanced nodes. SEP works in the same way as LEACH, but for SEP, the CH rotating epoch and election probability are directly related to the initial energy of nodes. However, for a multi-level heterogeneous WSN which are probably caused by the normal operations of a network or deploying new sensor nodes, SEP lacks corresponding considerations for residual energy.

As opposed to SEP, DEEC further improves the functions of election probability by considering both the initial and residual energy of the network. It achieves better performance than SEP and LEACH in a multi-level heterogeneous WSN. Unfortunately, DEEC can’t be used when the BS is located far from the sensor nodes since it is working under the assumption that BS is located in the center of the WSN. In addition, though modifying the election probability function can somehow guarantee that the nodes with relatively high initial and residual energy have larger probabilities to win the contest, some low-energy nodes still have a chance to be successfully elected as cluster heads.

Meanwhile, some protocols like EECS [19] and SEECH [20] introduce the concept of candidate CH nodes, and then choose the final CH set based on some other metrics. In EECS, nodes elect themselves to be the candidate nodes for CHs. Then, if a candidate node finds any more powerful candidate within a specific radio range, it will quit the competition, ensuring that high-energy nodes will be elected. 

SEECH innovatively proposed a protocol which selects CHs and relays separately. The reason for using relay nodes is to further mitigate the energy burden of CH nodes. The algorithm first selects some tentative cluster heads and then utilizes a specific probability to calculate the final CH set. Nonetheless, from the probability function, the priority level of a node is more heavily dependent on nodes degrees rather than residual energy. Thus, a low-energy node may still have a high possibility of being chosen as a CH node if it has a large degree.

As for the evaluation metric, we adopt the stability period to evaluate the performance of protocols since it can help to check whether the load of energy consumption is distributed evenly over the network. If we adopt the period of time until all nodes deplete their energy as the evaluation metric, some sensor nodes with higher energy will have great effects on the performance and the results may not reflect the working conditions of the whole network. The authors of [11] use the coverage rate as an auxiliary metric to evaluate their clustering protocol. This inspires us to consider introducing other metrics (e.g., coverage rate) to the existing metrics (e.g., stability period and network lifetime) in our further work, to better reflect the working conditions and monitoring quality of the whole network.

Our work is performed on the basis of DEEC and inherits the merit of considering energy heterogeneity, but two main modifications are made to promote the application scope: the first is that we consider situations where BS is located far from the sensor nodes, which is a typical scenario, but ignored by DEEC; the second is that we introduce a two-phase CH-election procedure to generate and replace tentative CH nodes, ensuring that nodes with more energy have higher probabilities of being the final cluster heads. The network model and assumption are described in the following section.

## 3. Heterogeneous Network Model and Assumptions

In this section, we first make several assumptions about the network model and explain them in detail. Then, at the end of this section, we summarize and list these assumptions, to make the design features more clear.

In our work, we consider a WSN consisting of N sensor nodes $s_{i}\;\left(i=1,2,\ldots ,N\right)$, which are uniformly dispersed over a target area with size W×W to continuously monitor the environment. For sensing large or hazardous areas, it is hard to conduct deterministic deployment, in which sensor nodes are set at the ideal locations manually. Thus, the assumption of random node placement is practical, and can be used to generate a general network topology for simulation.

The sensor nodes are stationary after deployment, and they always have data to transmit to a fixed BS, which is often far from the sensing area. In contrast to DEEC, we assume that the BS is located at the coordinate (0,H), if the center of the area is set as the origin point. In the following section, the coordinate of BS can be used to analyze and estimate the energy consumption of data transmission between CHs and BSs.

For the energy heterogeneity of sensor nodes, we assume that the initial energy of sensor nodes is randomly distributed between $E_{0}$ and $\left(1+a_{max}\right)E_{0}$. $E_{0}$ is the lower bound of energy, and $a_{max}$ is the heterogeneity factor which determines the maximum percentage of extra battery energy. Each sensor node has an initial energy of $\left(1+a_{i}\right)E_{0}$, meaning that it has $a_{i}$ times more energy than the reference energy level $E_{0}$. In addition, we assume that each node knows the overall energy of the network, which can be achieved by receiving a broadcast message from the BS. The total energy of whole heterogeneous WSN is as follows:

(1) $$E_{Total}=\overset{N}{\underset{i=1}{\sum }}\left(1+a_{i}\right)E_{0}=E_{0}\left(N+\overset{N}{\underset{i}{\sum }}a_{i}\right)=E_{0}\left(N+A\right)$$

In our network, sensor nodes are clustered into a hierarchy structure, and the CH nodes can aggregate the correlated data to discard duplicated information. In addition, we assume that the sensed data in the same cluster is highly correlated, and can be aggregated into a fixed-length packet. Each non-cluster head node sends $l_{D}$ bits of data to CHs per round, and the energy consumption rate for CHs to operate data aggregations is $E_{DA}$ (nJ/bit/signal). A broadcast packet containing control messages is $l_{C}$ bits.

For data transmission, we assume that the transmission link between CM and CH or CH and BS is one-hop and symmetric. We will consider a multi-hop network in our further work. We use a simplified model in [21] to estimate the communication energy consumption. Based on the distance between the transmitter and the receiver, the free-space or multi-path channel model are used. The required energy for transmitting a l-bit packet over distance d m is:

(2) $$E_{TX}=\{\begin{array}{c} lE_{elec}+l\varepsilon_{fs}d^{2}\;\;\;\;\;\;d\leq d_{break}\\ lE_{elec}+l\varepsilon_{mp}d^{4}\;\;\;\;\;d>d_{break}\; \end{array}$$

where $E_{elec}$, $\varepsilon_{fs}$ and $\varepsilon_{mp}$ are parameters of the transmission or reception circuits while $d_{break}$ is a threshold distance deciding the corresponding channel model. The energy for receiving l bits of data is:

(3) $$E_{RX}=lE_{elec}$$

Assuming that CH nodes can aggregate the data from its CM nodes into a $l_{D}$-bit packet, the total estimated energy dissipated in the network during a round is [15]:

(4) $$E_{Round}=l_{D}\left(2NE_{elec}+NE_{DA}+k\varepsilon_{m}d_{H2B}^{4}+k\varepsilon_{f}d_{M2H}^{2}\right)$$

where $d_{H2B}$ is the average distance between CH nodes and BS, and $d_{M2H}$ is the average distance between CM nodes and CH nodes. Note that Equation (4) is deduced in the ideal condition where each cluster has the same size, and thus it can be seen as the least average energy consumption for a network in a single round. Thus, we can estimate the lifetime of a network when knowing the total energy of the network and Equation (4). $d_{M2H}$ can be calculated from the following equation:

(5) $$d_{M2H}=\frac{W}{\sqrt{2\pi k}}$$

where k is the expected number of clusters. Since in the series of LEACH-like protocols, the optimal percentage of clusters is a pre-defined parameter $p_{opt}$. We can simply set $k=Np_{opt}$ and calculate $d_{M2H}$.

To sum up, some import assumptions we make in this section are listed as follows.

1. The BS is located far from the sensing area, and sensor nodes are stationary after random deployment.
2. The WSN is a heterogeneous one which means the initial energy of sensor nodes is different.
3. Sensor nodes are clustered into a hierarchy structure, in which the sensed data of CM nodes are gathered by CH nodes and sent to the BS after aggregation.
4. The communication links are one-hop and symmetric, and a message can be broadcast to all the nodes in the network.
5. Sensor nodes can estimate the distances to other nodes based on the received signal power, and they can adjust the transmission power according to the estimated distances to the recipients.

## 4. The DCE Protocol

**Figure 2:**
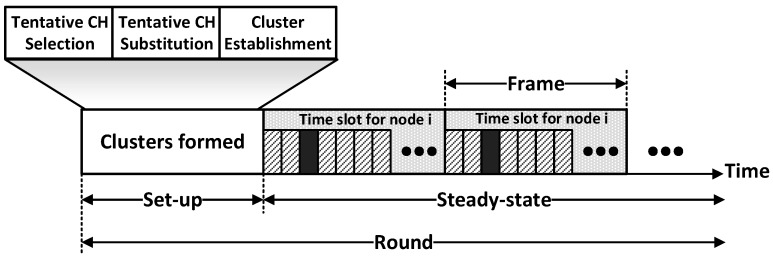
A timeline showing the operations of DCE: to form a cluster, the network goes through three main operations in the set-up stage, i.e., tentative CH selection, tentative CH substitution and cluster establishment; in the steady-state stage where data transmission is performed, the operation is divided into frames and each node transmits its data in assigned time slot.

In this section, we present the DCE protocol and analyze its characteristics. After distributing sensor nodes into a target area, the proposed DCE protocol begins to work. To be specific, DCE operates by round and each round includes the set-up stage and steady-state stage, as illustrated in Figure 2. The network initializes all the sensor nodes before the first round, by broadcasting a message which has information about the total energy of the network, synchronization clock and the order to start. Then, in each round, the protocol launches the set-up stage and the steady-state stage consecutively just like in other LEACH-like protocols. In the set-up stage, the network has two main operations which are cluster head election and cluster establishment.

The main difference between DCE and other LEACH-like protocols lies in the cluster head election procedure in the set-up stage. Depicted in Figure 2, the set-up stage is divided into three phases, among which tentative CH substitution is newly added. In the first phase, the proposed protocol selects tentative cluster heads based on probabilities that are decided by the relative levels of initial and residual energy of sensor nodes. Then, in the second phase, DCE decides the final set of cluster heads by replacing the low-energy tentative cluster heads with the more-powerful CM nodes. These two phases guarantee that nodes with more energy have a higher probability of being the cluster heads. Then, clusters are formally established in the cluster establishment phase. We will give the details of the first two phases in the following subsections.

### 4.1. Tentative Cluster Head Selection

In energy-homogenous WSN, LEACH tries to ensure the optimal percentage of cluster heads in the network is a fixed value, $p_{opt}$, by setting the rotating epoch for cluster heads to $\left\lceil 1/p_{opt}\;\right\rceil$. However, for reasons of networking evolution or deploying heterogeneous sensor nodes, the probability function and the rotating epoch in LEACH is no longer suitable for the new scenario.

Consequently, each sensor node should dynamically decide its own election probability $p_{i}$, by considering its initial and residual energy level. In DCE, we follow the modifications in DEEC to adjust the probability for a sensor node $s_{i}$ being a CH to

(6) $$p_{i}=p_{opt}\times \frac{\left(1+a_{i}\right)}{\frac{\left(N+A\right)}{N}}\times \frac{E_{i}\left(r\right)}{\bar{E}\left(r\right)}=\frac{p_{opt}N\left(1+a_{i}\right)E_{i}\left(r\right)}{\left(N+A\right)\bar{E}\left(r\right)}$$

where $E_{i}\left(r\right)$ is the residual energy of $s_{i}$ at round r, and $\bar{E}\left(r\right)$ denotes the average energy of the network at round r. $\bar{E}\left(r\right)$ can be calculate by

(7) $$\bar{E}\left(r\right)=\frac{1}{N}\overset{N}{\underset{i=1}{\sum }}E_{i}\left(r\right)$$

From Equation (6), we learn that the election probability $p_{i}$ consists of three parts: pre-defined $p_{opt}$, the relative level of initial energy, and the relative level of residual energy. $p_{i}$ fluctuates around $p_{opt}$ according to the two relative energy levels. Therefore, an individual sensor node $s_{i}$ can dynamically adjust its probability. When the relative levels of initial energy and residual energy of a sensor node are all higher than those of other nodes, it will have a much higher probability of being a CH. This condition usually happens at the beginning period of the network lifetime. In other conditions, the priorities of nodes are more complex, and need to be judged by Equation (6). Since both the initial energy and the residual energy are considered in the function of election probability, our proposed protocol reacts better and adjust faster, than the protocols like LEACH and SEP. 

In the tentative CH selection phase of each round, each node calculates its probability threshold, generates a random number between 0 and 1, and elects itself to be a tentative CH when the random number is smaller than the threshold. The probability threshold for $s_{i}$ is related with $p_{i}$ and given as follows

(8) $$T\left(s_{i}\right)=\{\begin{array}{cc} \frac{p_{i}}{1-p_{i}\left(r\;mod\;\lceil \frac{1}{p_{i}}\rceil \right)} & ,\;\mathrm{if}\;s_{i}\in C\\ 0 & ,\;\mathrm{if}\;s_{i}\notin C \end{array}$$

where C is the set of nodes that are eligible to take part in the election at round *r.* If $s_{i}$ has not been elected as a CH in the last $\left\lceil 1/p_{i}\;\right\rceil$ rounds, then $s_{i}\in C$.

As seen from Equation (8), the threshold not only correlates with $p_{i}$, but also with the remainder from dividing round *r* by $\left\lceil 1/p_{i}\;\right\rceil$. As a result, the threshold value will gradually increase if node $s_{i}$ hasn’t been a CH in the last $\left\lceil 1/p_{i}\;\right\rceil$ round. Hence, the threshold function tries to make the interval between two successfully-elected rounds in a reasonable range. In comparison to LEACH, the threshold function in Equation (8) is more adaptive to energy changes in the network.

### 4.2. Estimating Average Residual Energy

From Equation (7), we can find that the average energy $\bar{E}\left(r\right)$ is a parameter which needs global information about the total energy of a network. For a distributed protocol, it is burdensome work to realize. Thus, we intend to estimate $\bar{E}\left(r\right)$ by analyzing the average energy consumption of the network. Actually, $\bar{E}\left(r\right)$ is the ideal energy that each node should have in current round to maintain a good energy distribution. In ideal conditions, all the nodes deplete their energy at the same time, and the network achieves the longest stability period. The residual energy part in (6) not only controls the election probability of each node, but also the overall probability of the network. Accordingly, we estimate $\bar{E}\left(r\right)$ as follows

(9) $$\bar{E}\left(r\right)=\frac{1}{N}E_{Total}\left(1-\frac{r}{R_{opt}}\right)$$

where $R_{opt}$ represents the optimal round of the network lifetime. Since we have got $E_{Round}$ which represents the least average energy dissipated in the network per round in Section 3, $R_{opt}$ can be calculated by

(10) $$R_{opt}=\frac{E_{Total}}{E_{Round}}$$

Recalling Equation (4) in Section 3, we find that $d_{H2B}$, which is the average distance between CH nodes and BS, is still waiting for calculation. Unfortunately, LEACH ignored the estimation of this value while DEEC failed to consider the scenario where the BS is outside the deployment area of sensor nodes. Thus, we supplement this part in our protocol. The calculation procedure follows the same model and assumptions in Section 3. 

**Figure 3:**
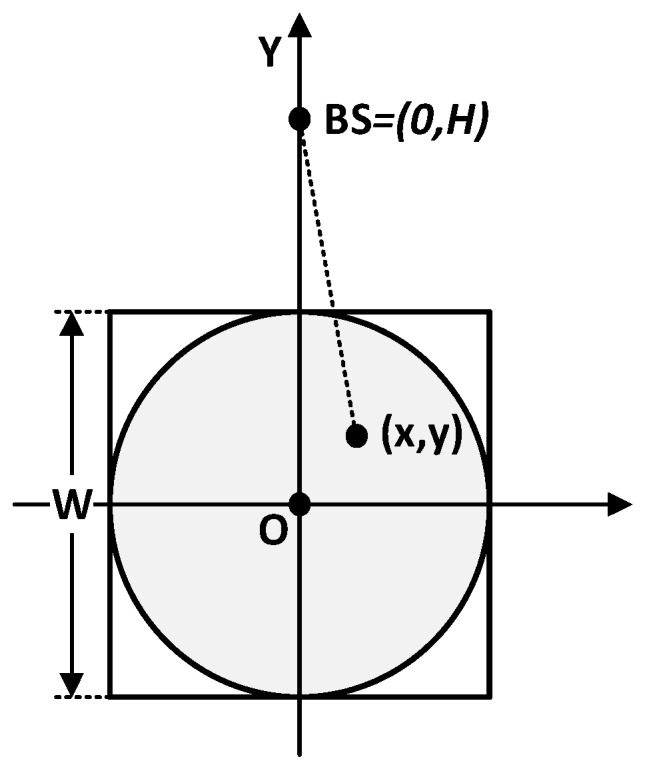
The diagram for calculating$\;d_{H2B}$, which is the average distance between CH nodes and BS.

Shown in Figure 3, the CH can be anywhere in the square area with side length W. We assume that the coordinate of a cluster head is (x,y) and the distribution function of a CH node is ρ(x,y). Since the density of the CH is uniform throughout the area, then $\rho \left(x,y\right)=\rho =1/W^{2}$. Then, the expected squared $d_{H2B}$ is given by

(11) $$\begin{array}{ll} E\left[d_{H2B}^{2}\right] & =\iint^{\text{​}}{\left(x-0\right)}^{2}+{\left(y-H\right)}^{2}\rho dxdy\\ & =\rho \iint^{\text{​}}x^{2}+y^{2}-2yH+H^{2}dxdy \end{array}$$

For the convenience of calculations, we assume that the CH lies inside the grey circle in Figure 3. By transforming Equation (11) into polar coordinates, we can get

(12) $$\begin{array}{ll} E\left[d_{H2B}^{2}\right] & =\rho \iint^{\text{​}}x^{2}+y^{2}-2yH+H^{2}dxdy\\ & =\rho \int_{0}^{2\pi }\int_{0}^{W/2}(r^{2}-2r\sin\theta H+H^{2})rdrd\theta \\ & =\frac{\pi \left(W^{2}+8H^{2}\right)}{32} \end{array}$$

Consequently, we get $d_{H2B}=\sqrt{\pi \left(W^{2}+8H^{2}\right)/32}$ from Equation (12). Since we have limited the location of the CH to the grey circle instead of the whole square area, some correction factor should be added to $d_{H2B}$. Through simulations, we can deduce that the actual distance is 1.12 times the value of $d_{H2B}$. Thus, we modify the value of $d_{H2B}$ to

(13) $$d_{H2B}=1.12\sqrt{\frac{\pi \left(W^{2}+8H^{2}\right)}{32}}$$

Now, utilizing the above parameters, we can now calculate the probability threshold of each node $s_{i}$ in Equation (8). However, there is a special case for the probability threshold when $\bar{E}\left(r\right)<0$. This case exists since the energy consumption of each node can’t go accurately as expected. To solve this issue, we can set $R_{opt}$ to a larger value [18] or make $p_{i}=p_{opt}$. We employ the latter one in our protocol.

Using the probability threshold, each node autonomously decides its election state through local calculations, and introduces itself to the whole network as a tentative cluster head if elected. The latter procedure is achieved by broadcasting a short message using a non-persistent carrier-sense multiple access (CSMA) media access control (MAC) protocol [22]. The message contains the node’s ID, the condition of residual energy, and a header which marks this message as an announcement to declare its state of being a tentative CH. After the deadline of the tentative CH election phase, the protocol goes into the next phase to check whether some low-energy tentative CHs can be replaced by those cluster members with more energy.

### 4.3. Replacing Low-Energy Tentative CHs

A considerable amount of research has been done on modifying the probability function to avoid low-energy sensor nodes being elected. Actually, selecting low-energy nodes as cluster heads may accelerate their death, which worsens the stability period of the network. However, it’s hard to predict the interactions between sensor nodes in a network with even a dozen of nodes. For reasons of network randomness and distributed design, the defect still exists. Thus, we propose to replace the low-energy tentative cluster heads to form an optimized set of final cluster heads, which further enhances the performance of our proposed protocol.

In this phase, each non-elected node determines its tentative cluster with the minimum communication energy, according to the received signal strength (RSS) of the announcement message from the tentative cluster heads. Then, the non-elected nodes compare their own residual energy with that of their cluster head. If a non-elected node possesses more energy, it will broadcast a short announcement message to the whole network, to state its replacement of the original CH node and its request to be the final CH node of this round. For the sake of avoiding the occurrence of multiple substitutions for the same tentative CH, all the nodes, which are eligible to conduct the substitution, broadcast the announcement message randomly within a bounded period of time. Once again, nodes sense the propagation channel and decide whether it can broadcast the message. Accordingly, the first node who successfully declares its substitution will be the final successor to original CH, while other eligible nodes intending to replace the same CH give up the competition and wait to join the cluster established by a new cluster head.

To be specific, the substitution of low-energy tentative cluster heads takes place between the operations of tentative CH election and establishing new clusters. This operation aims at further promoting the possibility of electing high-energy nodes to be the cluster heads. Then, in the operation of cluster establishment, each non-elected node sends a request message to join the nearest cluster, and cluster heads set up a time-division multiple-access (TDMA) [21] MAC schedule for the cluster members in their clusters. The steady-state works the same way as in LEACH protocol and the detailed description is omitted.

## 5. Simulations

In this section, we evaluate the performance of DCE protocol via extensive simulations in terms of the stability period. In general, a longer stability period represents a more balanced energy consumption in the network, which is also the primary target of our protocol. Note that the effects caused by signal collision and interference in the wireless channel are ignored. In the following text, we first give a general case in Scenario 1 to show the effectiveness of DCE, and then evaluate the effects of some important parameters (such as heterogeneity factor $a_{max}$, the predefined percentage of cluster heads $p_{opt}$, node number N and the location of BS) on stability period. Except for Scenario 1, the results of other scenarios are the statistical average of 100 simulations.

We compare the proposed DCE protocol with LEACH, SEP, DEEC, and DEEC-RE. Note that in our simulation, we follow the modifications to SEP in [18], to make SEP suitable for a multi-level heterogeneous WSN. DEEC-RE is a modified DEEC protocol which keeps the residual energy part in Equation (6), but holds other characteristics of DEEC. Indeed, evaluating the performance of SEP and DEEC-RE contributes to comparing the impacts of initial energy and residual energy.

### 5.1. A General Case of Simulation

In Scenario 1, we consider a WSN with N=100 which is randomly dispersed in a square area with W=100 m. For the energy heterogeneity, $a_{max}=1$. The BS is located at (0, 200) when setting the center of the area as the origin point. Other general parameters which are also used in this section are listed in Table 1. 

**Figure 4:**
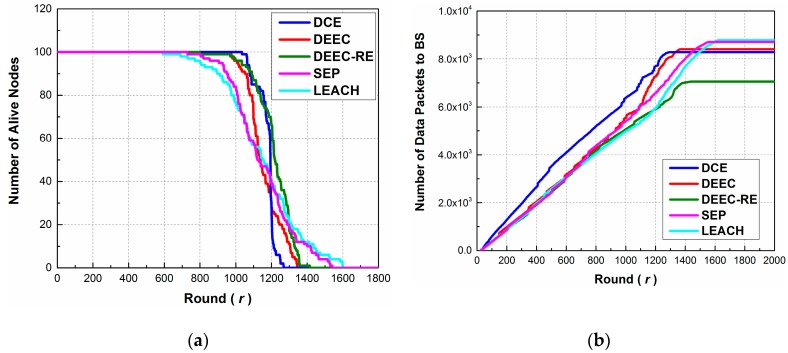
The performance of DCE, DEEC, DEEC-RE, SEP and LEACH in Scenario 1: (**a**) Number of Alive nodes by round; (**b**) Number of data packets to BS by round.

As we can observe from Figure 4a, the stability period of DCE is 1034 rounds, which is the longest among all the protocols. DEEC, DEEC-RE, SEP and LEACH achieve stability periods of 973, 797, 729 and 590 rounds, respectively. Compared to DEEC, the performance of the proposed protocol has risen by 6.3%. To be specific, SEP outperforms LEACH since SEP takes the impact of initial energy into consideration; DEEC is superior to DEEC-RE and SEP because it considers both residual energy and initial energy; DEEC-RE performs better than SEP because it adapts to energy change; DCE performs best since it has an extra phase for replacing the low-energy cluster heads, avoiding those nodes being elected. In addition, after the stability period, DCE dies much faster than other protocols. The reason lies in the fact that DCE maintains a better distribution of energy consumption, which means that sensor nodes exhaust their energy almost in the same period of time. The instability period of LEACH lasts longer, since some high-energy nodes are still alive after the first node dies. 

On the other hand, Figure 4b shows the relations between the number of data packets sent to BS by operation round. The packet numbers of all the protocols have an upward trend at first, and remain stable after there are no more alive nodes. Setting the 1304th round (the stability period of DCE) as a reference time point, we can see that cluster heads send more packets to the BS in DCE than other protocols, implying that DCE achieves higher energy efficiency than other protocols. Although some protocols surpass DCE in the end, the information extracted from the packets received by BS is somewhat incomplete, since just few nodes are alive and end-users lose contacts with most nodes at this time.

The results above show the effectiveness of DCE, and indicate the importance of designing mechanisms to ensure choosing high-energy nodes as cluster heads.

### 5.2. The Effect of Heterogeneity Factor

In Scenario 2, we consider evaluating the effect of energy heterogeneity on the stability period of the network. Since the proposed protocol works in heterogeneous WSNs, the robustness to energy heterogeneity is of great importance. In this scenario, we set the total energy of the network as 75 J, which is the same as that in Scenario 1. Additionally, $a_{max}$ ranges from 0 to 7 with 1 as the increment.

**Figure 5:**
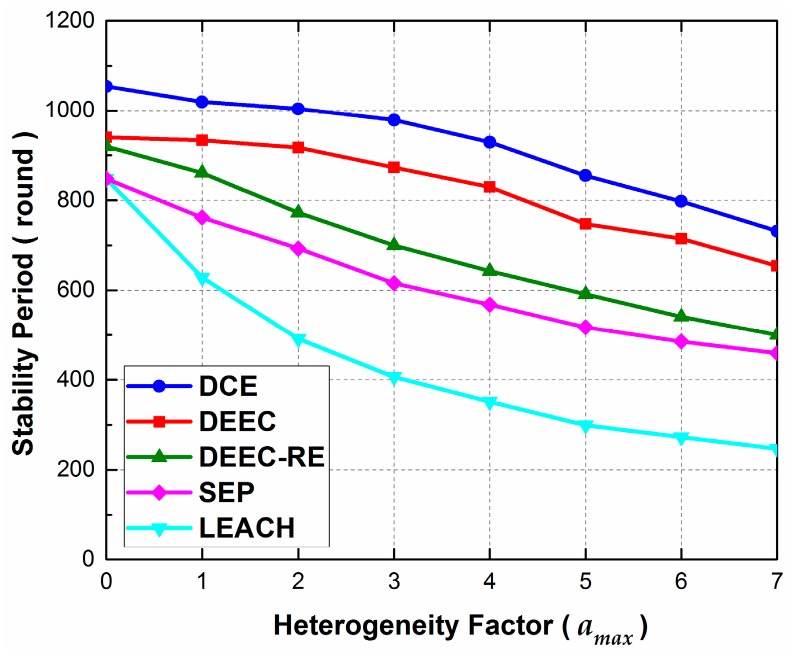
The effect of heterogeneity factor $a_{max}$ on stability period in Scenario 2. Note that, in this scenario, the total energy of the network is the same as that in Scenario 1, and just the energy heterogeneity is changed.

As we can observe from Figure 5, the stability periods of all protocols decrease monotonically with $a_{max}$. The reason is that the distributed protocols can’t fully optimize network performance, and severe energy heterogeneity worsens the condition of energy load distribution, which is reflected in stability time. Nonetheless, it should be noted that we are exploring and emphasizing the effect of energy heterogeneity, instead of comparing distributed and centralized designing. Moreover, the proposed protocol performs better than other protocols when changing $a_{max}$. When $a_{max}=0$, which means the WSN turns back into a homogeneous one, the performance of DEEC and DEEC-RE are almost the same, and this is also true for LEACH and SEP. The reason lies in the fact that the parts of initial energy level in the election probability function lose their meaning when no energy heterogeneity exists. However, with the increase of $a_{max}$, the stability period of LEACH drops much faster than other protocols, and the performance gap between DEEC and DEEC-RE increases as well. Thus, we may infer that, both the initial energy and the residual energy have effects on the stability period, and they should be taken into account when designing the function of election probability.

### 5.3. The Effect of Predefined CH Percentage

In Scenario 3, the effects of the predefined percentage of cluster heads $p_{opt}$ is evaluated. The simulation parameters are the same as those in Scenario 1, apart from that $p_{opt}$ is changed from 0 to 0.09 with 0.01 as the increment.

**Figure 6:**
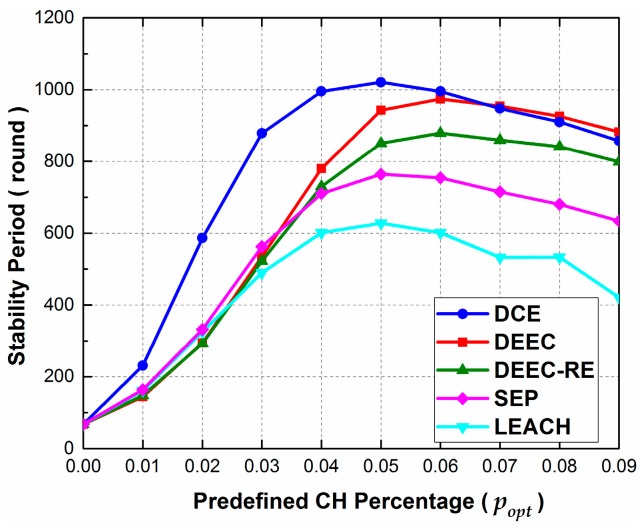
The effect of predefined percentage of cluster heads $p_{opt}$ on stability period in Scenario 3.

Figure 6 shows how the stability period changes with the increase of $p_{opt}$. From the figure, we observe that the stability period of all protocols have a rapid growth at first, and peak at $p_{opt}=\;$0.05 or $p_{opt}=0.06$. Then, the stability periods decline gradually with the increase of $p_{opt}$. The reason is that setting an appropriate percentage of cluster heads helps to reduce the quality of data sent to BS by data aggregation and therefore lessens average energy consumption per round.

When $p_{opt}=\;$0.00, sensor nodes in all the protocols transmit the sensed data to BS directly and no data aggregation is conducted, since there is no cluster head. In this condition, all the protocols achieve equal and minimum rounds of stability period. Then, we can find that the peak value of DCE outperforms those of others, since DCE has an extra phase to replace the inaccurately-chosen cluster heads. The values for stability period go down after achieving peak values, because data aggregation is not sufficient when choosing additional cluster heads. Here, an extreme case is when $p_{opt}=\;$1.00, which means almost all the nodes can be chosen as cluster heads. Under this condition, since all the sensors can be cluster heads and there is nearly no cluster member, the results of stability period can be as bad as those achieved when $p_{opt}=\;$0.00. Additionally, we can find that the results of DEEC are slightly superior to those of the proposed protocol when $p_{opt}\geq$ 0.07. This is due to the extra energy consumption brought by the phase of replacing low-energy tentative CHs.

Since the predefined CH percentage directly affects stability period, it is significant to choose proper value of $p_{opt}$ before executing the protocols. In the next subsection, we investigate the effects of node number and BS location on $p_{opt}$ and stability period.

### 5.4. The Effect of BS Location and Node Number

In this section, we evaluate the effects of altering BS location and node number N in Scenario 4 and Scenario 5 respectively. The purposes of this subsection are twofold: to evaluate the effect of these two parameters on stability period, and to give some guidelines for choosing proper$\;p_{opt}$.

**Figure 7:**
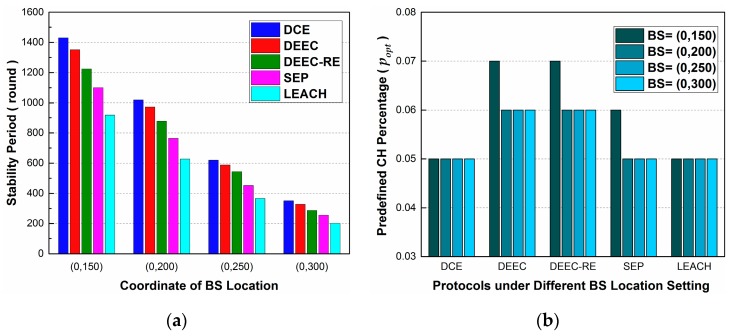
The effect of altering BS location on stability period in Scenario 4: (**a**) The best stability period achieved under different settings of BS location; (**b**) The corresponding $p_{opt}$ where the longest stability period is achieved.

In Scenario 4, the parameters for simulations are the same as those in Scenario 1, except that the coordinates of BS locations are changed from (0, 200) to (0, 150), (0, 250) and (0, 300). In addition, the stability period in the following results is the longest average stable time that each protocol can obtain under different settings of $p_{opt}$. To be specific, since we don’t have the prior knowledge about how to choose exact values of $p_{opt}$ under different settings of BS location, we conduct dozens of simulations by changing $p_{opt}$ from 0.00 to 0.10 at different BS locations. Then, for a specific protocol at a specific location, we choose the result with the longest average stable time and present it Figure 7a. The corresponding values of $p_{opt}\;$are presented in Figure 7b.

Observing Figure 7a, we can know that, as the BS is getting far away from sensor node area, the rounds of stability period in all the protocols decrease but DCE always performs better than others. It is obvious that the farther the BS is, the more energy will be consumed per round in WSN, leading to shorter stability periods. In Figure 7b, the corresponding $p_{opt}$, where the longest stability period is achieved, is presented. From the statistics, we find that the $p_{opt}$ for achieving the longest stability period almost remains the same when changing BS location, meaning that BS location has little direct impact on setting $p_{opt}$. Note that some differences occur in the results of BS=(0, 150) for DEEC, DEEC-RE and SEP in Figure 7b. The reason may lie in the fact that a sensor node always considers communicating with the nodes within the distance of $d_{break}$, since this way is more economical from its own perspective. When $d_{break}=87.7\;m$ in our initial settings, some nodes near the upper boundary of the sensing area choose to communicate with the BS directly, instead of joining clusters, which adds extra energy burdens to CH nodes. In the other three cases, this phenomenon doesn’t appear, since the distances to the BS are larger than $d_{break}$. Therefore, there is some difference at location (0, 150), but as a whole, $p_{opt}$ almost remains the same. Thus, we don’t have to change the parameter of $p_{opt}$ in practical applications where just the location of BS is changed.

**Figure 8:**
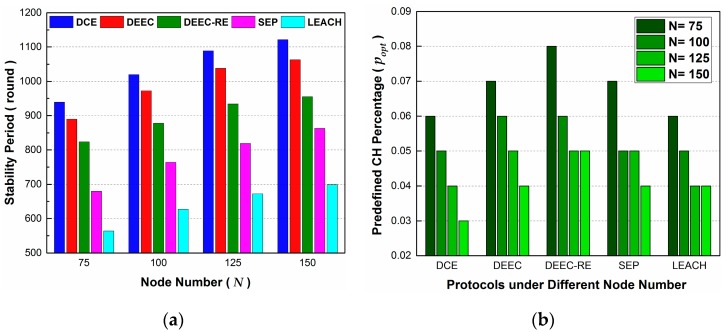
The effect of node number on stability period in Scenario 5: (**a**) The best stability period achieved under different N; (**b**) The corresponding $p_{opt}$ where the best stability period is achieved.

Next, the simulations in Scenario 5 are quite like those in Scenario 4, but here we evaluate the effect of changing node number N. In the simulations, N is changed from 75 to 150, with 25 as the increment. Figure 8a shows that, with the growth of N, the values for stability period in all protocols increase and DCE outperforms others. The burden of being cluster heads can be shared by more nodes, in the context of a larger quantity of sensor nodes in a network. Thus, energy consumption is better distributed over network, and the stability period is extended. DCE surpasses others due to its extra design for guaranteeing the selection of high-energy nodes as cluster heads.

In Figure 8b, for each protocol, the corresponding $p_{opt}$ has a downward trend with the increase of N. As we can know from Section 5.3, the stability period has an upward trend first, peaks at a specific $p_{opt}$ value, and then declines gradually with the increase of $p_{opt}$. At the point of peak value, a protocol achieves the maximum efficiency, since it maintains an efficient data aggregation. When we increase N and keep $p_{opt}$ the same, the average distances between CH and BS remain unchanged, and those between CH and CM declines. Although these lead to a decrease in the average energy consumption of a cluster per round, there are more clusters in the network and data aggregation is not the most efficient as well. On the other hand, if we set lower values for $p_{opt}$ when increasing N, the protocol may have a chance to conduct more efficient data aggregations, which can reduce the total quantity of data. In addition, although the energy consumption for being a cluster head increases due to having more data to handle, the possibility of a node being successfully elected as a CH declines, and the energy consumption is well distributed over the network. When the deployment area is fixed, larger node numbers means greater node density. Thus, we should adjust the value of $p_{opt}$ when applying the protocols to a WSN with different node density.

Through the simulations in this subsection, we find that it is node number, instead of BS location, that affects the setting of $p_{opt}$ when keeping other parameters unchanged. Since $p_{opt}$ is related with the longest stability period of the network, it is necessary to choose proper values for it. Actually, there are relevant analyses about calculating the predefined optimal percentage of cluster heads in [4], and in their opinion, the average distance from CH to BS affects network lifetime. However, from the simulations above, this conclusion seems no longer valid for our heterogeneous WSN. Thus, parts of the analyses in [4] can’t be used directly in the new scenario.

Unfortunately, a limitation of our current work is that no analysis formula for calculating $p_{opt}$ has been found yet and some further research work should be done on this issue. Notwithstanding the limitation, this study does demonstrate the importance of reasonably setting up the predefined parameters like $p_{opt}$ to promote the performance of stability period.

## 6. Conclusions

In this paper, we propose and evaluate a novel distributed energy-efficient clustering protocol called DCE for heterogeneous wireless sensor networks. The innovation of DCE lies in that we introduce an extra phase for cluster head election, which can prolong the stability period of the network. In our protocol, sensor nodes in the network first autonomously elect tentative cluster heads, by using a probability function which is decided by the relative levels of initial and residual energy. Then, in the newly-introduced substitution phase, the tentative cluster heads are replaced by those cluster members which have more residual energy, to form the final set of cluster heads. These phases guarantee that the nodes with more energy have more chance to be cluster heads, and the load of energy consumption is well-distributed over the whole network. Through extensive analysis and simulations, we found that DCE outperforms other algorithms in terms of stability period. In addition, we also deduced that the predefined cluster-head percentage correlates with the node number of a network, and this contributes to determining a proper number of cluster heads for a network.

## Figures and Tables

**Table 1:** General parameters for simulations.

Parameter	Value	Parameter	Value
$E_{0}$	0.5 J	$\varepsilon_{fs}$	10 pJ/bit/$m^{2}$
$E_{elec}$	5 nJ/bit	$\varepsilon_{mp}$	0.0013 pJ/bit/$m^{4}$
$E_{DA}$	5 nJ/bit/message	$l_{D}$	4000 bits
$d_{break}$	87.7 m	$l_{C}$	16 bits
